# Strengths and limitations of non-disclosive data analysis: a comparison of breast cancer survival classifiers using VisualSHIELD

**DOI:** 10.3389/fgene.2024.1270387

**Published:** 2024-01-29

**Authors:** Danilo Tomasoni, Rosario Lombardo, Mario Lauria

**Affiliations:** ^1^ Fondazione the Microsoft Research–University of Trento Centre for Computational and Systems Biology (COSBI), Rovereto, Italy; ^2^ Department of Economics, University of Verona, Verona, Italy; ^3^ Department of Mathematics, University of Trento, Povo, Italy

**Keywords:** computational biology, transcriptomic data, transcriptional signatures, federated databases, non-disclosive data analysis

## Abstract

Preserving data privacy is an important concern in the research use of patient data. The DataSHIELD suite enables privacy-aware advanced statistical analysis in a federated setting. Despite its many applications, it has a few open practical issues: the complexity of hosting a federated infrastructure, the performance penalty imposed by the privacy-preserving constraints, and the ease of use by non-technical users. In this work, we describe a case study in which we review different breast cancer classifiers and report our findings about the limits and advantages of such non-disclosive suite of tools in a realistic setting. Five independent gene expression datasets of breast cancer survival were downloaded from Gene Expression Omnibus (GEO) and pooled together through the federated infrastructure. Three previously published and two newly proposed 5-year cancer-free survival risk score classifiers were trained in a federated environment, and an additional reference classifier was trained with unconstrained data access. The performance of these six classifiers was systematically evaluated, and the results show that i) the published classifiers do not generalize well when applied to patient cohorts that differ from those used to develop them; ii) among the methods we tried, the classification using logistic regression worked better on average, closely followed by random forest; iii) the unconstrained version of the logistic regression classifier outperformed the federated version by 4**%** on average. Reproducibility of our experiments is ensured through the use of VisualSHIELD, an open-source tool that augments DataSHIELD with new functions, a standardized deployment procedure, and a simple graphical user interface.

## Introduction

The data-driven research paradigm supported by large datasets and collaborative initiatives has been successful, but sharing medical data requires preserving patient privacy and respecting the legitimate concerns of authors about intellectual ownership. DataSHIELD (Wolfson et al., 2010; Gaye, 2014) was introduced to perform advanced statistical analysis across multiple, remotely hosted datasets without sharing any individual-level data. To the best of our knowledge, DataSHIELD is the only community-driven open-source framework for privacy-aware data analysis currently available. The data analysis is conducted in compliance with the legal and ethical regulations of the data-hosting country as the data never leave the institution where it was created. Only non-disclosive summary statistics computed at each data center are sent to the remote research institution that requested the analysis where they are aggregated to produce the final result. In this way, DataSHIELD combines the privacy of non-disclosive summary statistics with the aggregation of data kept at geographically remote federated institutions.

**FIGURE 1:**
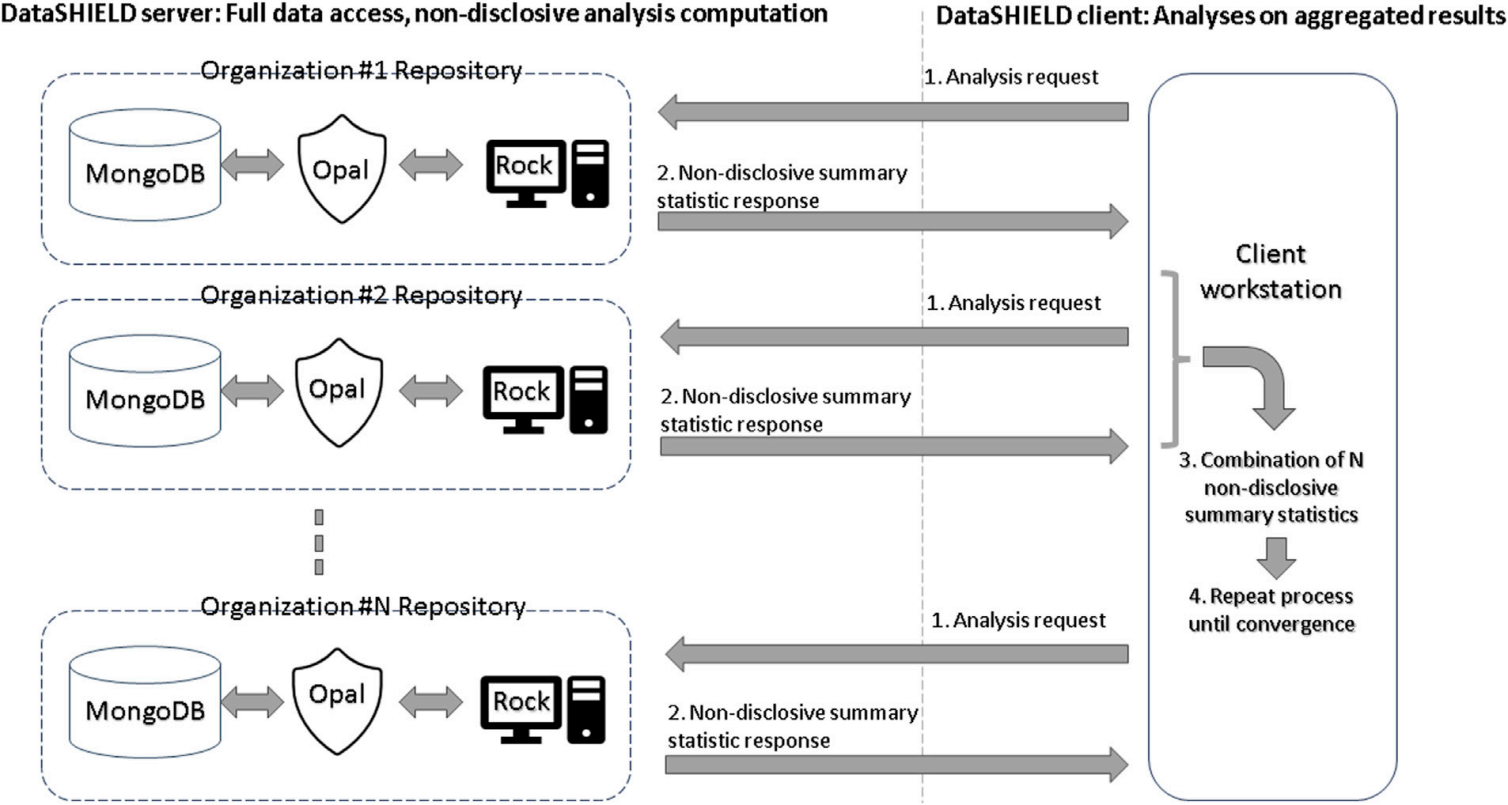
Summary of the DataSHIELD privacy-preserving federated framework.

In the DataSHIELD architecture, each data center hosts a database such as MongoDB, an Opal data store, and a Rock analysis server. Opal is OBiBa’s (Doiron et al., 2017) core data warehouse and provides all the necessary tools to import, transform, and describe locally stored observational data. Rock is OBiBa’s analysis server, enabling the execution of non-disclosive statistical analyses. DataSHIELD orchestrates the interaction between Opal and Rock in order to carry out federated non-disclosive algorithms. Figure 1 provides a summary of the DataSHIELD privacy-preserving federated framework. An undesirable side effect of such a multi-site architecture is that variations in the deployment of the infrastructure, due to different software versions and their interactions, can negatively impact the reproducibility of analyses performed on it.

Recent extensions to the original DataSHIELD suite have been published, resulting in a progressive increase in its functionality and ease of use. The “resources” architecture (Marcon et al., 2021) extends the functionality to the handling of data type beyond those for which the platform was originally designed. ShinyDataSHIELD (Xavier et al., 2022) addresses the ease-of-use concern with a Shiny app (Rstudio, 2013) that allows researchers to perform many types of statistical analyses without using the R command line. None of these extensions address the need for an easy and standardized deployment for researchers, consortiums, and system administrators (Data sharing in the age of deep learning 2023).

These and other tools were deployed, integrated, and harmonized within the Nutritional Phenotype Database in the European Nutritional Phenotype Assessment and Data Sharing Initiative (ENPADASI) project (Pinart et al., 2018a; Vitali, 2018) involving 51 institutions in nine countries, with the aim of building a decentralized infrastructure to store, query, and share observational data and metadata on research nutrition interventions.

As participants of the ENPADASI initiative, we were able to directly observe and collect specific concerns raised within the research community that need addressing for the tool to achieve its full potential. One such concern is the overall complexity from an end-user point of view: federated analyses in DataSHIELD require proficiency in R programming, while the sharing of data requires system administration skills in order to set-up a complex multi-site infrastructure (see Figure 1). Furthermore, the required federated-aware functionality is scattered across a number of packages: federated randomForest, K-nearest neighbors, and principal component analysis are available through a dedicated R package, dsSwissKnife (Dragan et al., 2020), while visualization of the results requires other R packages such as randomForestExplainer (Paluszynska et al., 2020), ggpubr, cowplot, and ggplot2. In view of these concerns, we were unable to find any previous work providing a satisfactory analysis of the issues of usability, reproducibility, and/or functionality disadvantages one can expect from using a non-disclosive federated framework.

In an attempt to address the usability and reproducibility issues, we developed VisualSHIELD, an open-source, extensible web interface that simultaneously provides a standardized deployment of the DataSHIELD infrastructure and a graphical user interface to dsSwissKnife and other R packages in order to simplify the definition of an analysis workflow and the visualization of the results.

Then, using VisualSHIELD, we aimed to characterize the functional penalty of a federated analysis, by selecting a representative case study and comparing the results obtained in the federated setting with those obtained in an unconstrained traditional analysis.

The case study we selected was the identification of breast cancer relapse based on patient expression profiles. Correct identification of the patient’s risk of recurrence ensures she receives adequate therapy, and to this aim, multiple prognostic classifiers have been proposed to determine the 5-year cancer-free survival probability (Huang et al., 2013; Skye Hung-Chun et al., 2017; Chen et al., 2021). However, results on this topic are known to suffer from a lack of reproducibility. A convincing validation for such a classifier would require the measurement of its accuracy on a sufficiently large and heterogeneous collection of datasets, and we reasoned that this would represent an ideal application for a federated collection of data repositories. Motivated by these considerations, we reviewed a set of previously published breast cancer prognostic classifiers using a collection of multi-cohort expression profiles that differ from those originally used by the respective authors. Additionally, we compared the results of the distributed analysis with those that would be obtained in a conventional setting, in which the same data held on a single machine are processed using conventional R packages, and by difference, we characterized the federated computation performance and functional penalty.

The main contributions of this work are as follows: first, we address the usability aspect by describing the VisualSHIELD approach to friendly GUI design and making available an open implementation of the tool. Then, we address the manageability and reproducibility aspect by providing scripts to automatically perform the installation of a minimal, standardized, and fully functional reference system to simplify the initial deployment by researchers and system administrators. Lastly, we characterize the performance penalty induced by the non-disclosive constraints using a mix of previously published and novel breast cancer prognostic classifiers and discuss the clinical applicability of such models.

## Methods

The VisualSHIELD tool (Tomasoni and Lombardo, 2024) was implemented as a Shiny module (Rstudio, 2013), a graphical R package that can be embedded into any user-defined Shiny app to provide the federated analysis capability. It was designed with an open-source architecture that makes it extensible and provides a clear framework for the addition of user-defined federated analyses.

The tool provides a simple graphical user interface that integrates DataSHIELD analysis methods such as histograms, contour plots, heatmaps (Supplementary Figure S1), boxplots, correlation matrix, generalized linear models (GLMs) (Supplementary Figure S2), and dsSwissKnife methods, such as K-nearest neighbors, principal component analysis, and randomForest.

Furthermore, we added an interactive feature selection functionality that fits a single-gene logistic model for all the genes in a list and collects the respective beta coefficients and the p-values, which can be downloaded and visualized as a volcano plot (Supplementary Figure S3).

A novel interactive linear regression functionality was implemented in VisualSHIELD by augmenting the GLM functionality with some statistics not available in DataSHIELD such as *R* ^2^, adjusted *R* ^2^, and F-score (Supplementary Figure S2).

Logistic regression models (henceforth referred to as logit) are a special case of generalized linear models (Nelder and Wedderburn, 1972), abbreviated GLMs. They are widely used in medicine to measure the association between the occurrence of an event (such as 5-year cancer-free survival), called the dependent variable, and a set of independent variables (such as the expression level of a set of genes). A number of medical prognostic tools using logistic regression-based classifiers have been proposed (Biondo et al., 2000; Kologlu et al., 2001).

In logistic regression, the dependent variable is forced to take a value in the interval [0, 1], which can be interpreted as the probability of the outcome being assigned to one of two groups, such as alive/not-alive 5-year survival status.

To evaluate the performances of the classifiers under consideration, we used the area under the receiver operating characteristic (AUROC) (Hastie et al., 2017). The ROC curve is a widely used plot that illustrates the performance of a classifier, measured using a two-valued metric, such as true positive rate (TPR)/false positive rate (FPR).

In a context like ours, where we need to compare different curves, the area under the ROC curve provides a single value that simplifies the comparison.

In order to provide a robust estimate of each method’s performance, we use the well-known cross-validation procedure (Hastie et al., 2017). Every classifier analyzed in this paper was trained five times, by using four out of five datasets as the training set and the remaining dataset as the test set. This method allows us to test the classifier performance stability when trained with different data. This version of the procedure is often referred to as the leave-1-dataset-out cross-validation.

In this work, we evaluate the performances of five different models using data stored in a non-disclosive federated environment, plus an additional “full” reference model validated with unrestricted access to locally stored data; all the models are designed as prognostic classifiers to predict the probability of breast cancer-free survival at 5 years using gene expression data.

Three of the five models are previously published GLMs; the remaining two are newly proposed models, specifically a GLM and random forest (Ho, 1995), which is abbreviated RF. The additional “full” reference model trained with full data access is also a GLM.

The three published GLMs were trained with the genes identified by the respective authors, while the two novel GLM and the RF models proposed in this paper were built starting from a small subset (262 genes) extracted from the full list (13,041 genes) of genes available in the profiles.

The subset was identified with the feature selection functionality available in VisualSHIELD, by fitting a single-gene logistic regression model for all the gene profiles in the datasets and collecting the beta coefficient and the p-value assigned to the gene by the respective model. The beta coefficient of the independent variable measures the change in the dependent variable for a unit change in the independent variable. Thus, independent variables with higher beta coefficients have a stronger influence on the dependent variable. The p-value estimates how likely it is to observe an association of the same or higher strength purely by chance.

Using these two measures, a volcano plot was constructed (Supplementary Figure S3), and only the significant genes (*p* < 0.05) with the most extreme beta values (below 1 and above 99 percentile) were retained.

Our two newly proposed logistic regression models are named “full” and “restricted.”

The ‘full’ model was obtained with a forward stepwise variable selection method (Hastie et al., 2017) applied to the 262 genes and all their interaction terms, accessing the data in a federated setting via VisualSHIELD. We implemented the stepwise selection because we were unable to find a federated implementation. The final model includes single genes and interaction terms for a total of 25 terms. Since the DataSHIELD-federated GLM implementation prevented us from running the “full” model in a leave-1-dataset-out setting (on the grounds that the model is disclosive of individual-level data when used on a small number of datasets), we derived a “restricted” version that was considered non-disclosive. “Restricted” was obtained starting from “full” by removing each gene/interaction term in turn, starting with the ones with the largest significant p-values and lower beta coefficients until it became acceptable from a non-disclosive point of view, according to the DataSHIELD criteria.

The random forest model was similarly trained on the list of 262 genes, again accessing the federated collection of five datasets through VisualSHIELD.

**FIGURE 2:**
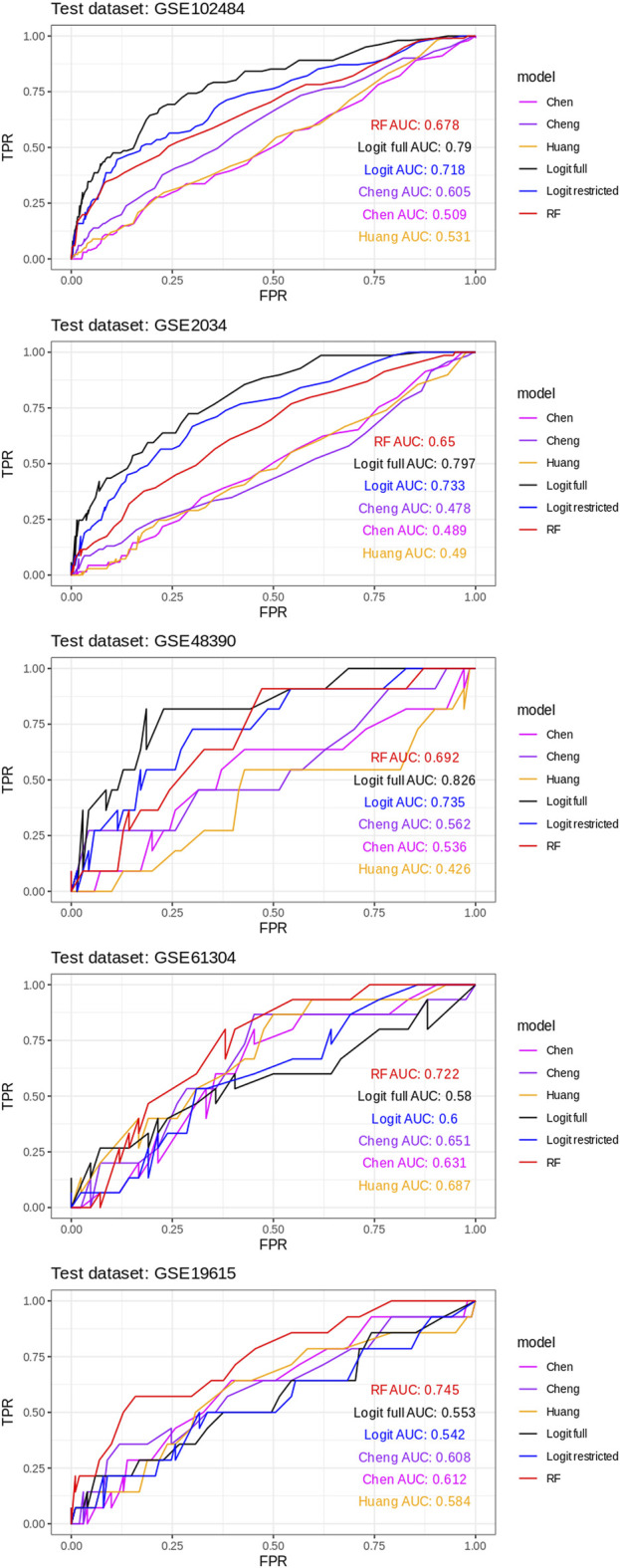
AUROC curves for all six classifiers for every train/test round using a leave-1-dataset-out validation scheme.

AUROC curves were estimated for all six classifiers for every train/test round of a leave-1-dataset-out validation scheme (Figure 2).

With respect to the actual implementation of the analyses described above, we followed a hybrid approach: the feature selection and training phase were executed through the VisualSHIELD web interface in a federated environment, except for the “full” model, that was built in a local R environment with the *glm* function. The test phase, the ROC curve analysis, and the plots were generated in a local R environment, by loading the models previously exported from VisualSHIELD to a file in the *rData* format and by direct use of the “full” model.

The five logistic regression-based classifiers were trained with the federated version of the GLM using *logit* as the link function.

The random forest (RF) model was developed using the dsSwissKnife function *dssRandomForest* (Dragan et al., 2020) that implements a federated version of the algorithm and is built on top of the *RandomForest* R package.

*Docker* (Merkel, 2014) is a tool used to automatically configure and run complex software infrastructures across very different machines. This technology has been used in VisualSHIELD to allow researchers to quickly configure and run the full DataSHIELD infrastructure (Figure 1) ready to be connected to the VisualSHIELD GUI for data analysis. Setting up this instance requires the researcher only to install Docker and run a single set-up command.

The five datasets used are publicly available for download in Gene Expression Omnibus (Edgar et al., 2002), abbreviated GEO, and contain independent gene expression datasets of breast cancer survival. They were harmonized with a custom R script and then imported in the Opal instance created through the VisualSHIELD Docker images.

We choose datasets created at different institutions at different times to maximize between-dataset diversity. They all share a phenotypic column with the 5-year cancer-free survival status and a large common set of gene expression profiles, which is used for prediction.

All the datasets come from gene expression studies using the DNA microarray platform Affymetrix Human Genome U133 Plus 2.0, except that GSE2034 used its predecessor, the Affymetrix Human Genome U133A (AffyMetrix datasheet 2023).

Raw probe ids were mapped to official gene symbols through the R AnnotationDbi suite of packages (Pagès et al., 2023), de-duplicated, and the raw profiles were normalized by subtracting the mean and dividing by the standard deviation.

The data were then imported into different Opal servers for the subsequent federated analyses. The five datasets we used are the following:

• **GSE102484 (Cheng et al.)**: this contains breast cancer samples obtained from 683 patients, originally used to develop a 18-gene prognostic classifier to estimate distant metastasis risk.
• **GSE2034**: this contains samples obtained from 286 lymph node negative patients, used to develop a 76-gene signature to identify patients who developed distant metastases within 5 years.
• **GSE48390 (Huang et al.)**: this contains breast cancer samples obtained from 81 Taiwanese women, used to develop a breast cancer relapse risk model based on 16 genes.
• **GSE61304**: this contains samples from 57 patients used to entail the novel bio-marker discovery of tumor aggressive grade (TAG) signature genes that allowed developing a novel patient prognostic grouping method selecting the 12 survival-significant sense–antisense (SA) gene pairs (SAGPs).
• **GSE19615**: this contains samples obtained from 115 patients from which the authors identified a small number of overexpressed and amplified genes from chromosome 8q22 that were associated with early breast cancer recurrence.

The three published classifiers, henceforth called Chen, Huang, and Cheng, from the names of their respective first authors, are the following:

• Chen et al. (2021) proposed a 20-gene classifier for predicting patients with a high/low risk of breast cancer recurrence within 5 years and validated it on a dataset of Asian breast cancer patients.
• Skye Hung-Chun et al. (2017) proposed and validated an 18-gene classifier to predict 5-year distant metastasis risk and defined a threshold score for high/low-risk patients. The primary outcome was the 5-year probability of freedom from distant metastasis (DMFP). In this paper, we repurposed this model to predict 5-year disease-free survival by equating absence of metastasis to disease-free survival.
• Huang et al. (2013) derived signatures associated with clinical ER and HER2 status and disease-free survival. Furthermore, they derived a 16-gene model to identify patients with a low risk for 5-year recurrence on a dataset of Han Chinese breast cancer patients that we used for our analysis.

The independent variables for all the logistic regression models under consideration are summarized in Table 1.

**TABLE 1:** Summary of the classifiers based on logistic regression (logit). For all models, the dependent variable (not shown for brevity) is the 5-year cancer-free survival status. The remaining classifier (not shown) is based on a random forest model, and it includes all the 262 genes resulting from the initial feature selection.

Name	Formula	Reference
Chen	BLM + BUB1B + CCR1 + CKAP5 + CLCA2 + DDX39 + DTX2 + ERBB2 + ESR1 + MKI67 + OBSL1 + PGR + PHACTR2 + PIM1 + PTI1 + RCHY1 + SF3B5 + STIL + TPX2 + YWHAB	PMC8010242
Huang	RCAN3 + MCOLN2 + DENND2D + RWDD3 + ZMYM6 + CAPZA1 + TRIM45 + GPR18 + WARS2 + SCRN1 + CSNK1E + HBXIP + MRPL20 + CSDE1 + COL20A1 + IKZF1 + batch	PMC3789693
Cheng	TRPV6 + DDX39 + BUB1B + CCR1 + STIL + BLM + C16ORF7 + PIM1 + TPX2 + PTI1 + TCF3 + CCNB1 + DTX2 + ENSA + RCHY1 + NFATC2IP + OBSL1 + MMP15	PMC5590926
Logit full	BTN2A2 + ALDH3B2 + EML1 + FKBP5 + IGFBP6 + LRRC32 + STX5 + RABAC1 + BCAM + TFIP11 + PLIN3 + SGK1 + TXNRD1 + PPP1CC + KATNBL1 + CHPT1 + IMP3 + GLUD1:SLC26A3 + SLC26A3:NDUFB1 + RLN1:RLN2 + SREK1:GORASP1 + SREK1:NTM + RLN1:RECQL4 + TXNIP:GORASP1 + PHF10:POLQ	New
Logit restricted	EML1 + IMP3 + BTN2A2 + FKBP5 + IGFBP6 + LRRC32 + STX5 + RABAC1 + TXNRD1 + KATNBL1 + GLUD1:SLC26A3 + SLC26A3:NDUFB1 + RLN1:RLN2 + SREK1:GORASP1 + TXNIP:GORASP1 + PHF10:POLQ + SREK1:NTM	New

## Results

We analyzed five independent gene expression datasets of breast cancer patients from Gene Expression Omnibus (GEO), collected from different times and different institutions to maximize the between-dataset diversity. We harmonized and pooled them together through the federated infrastructure.

They all share a phenotypic column with the 5-year cancer-free survival status that was used to train six prognostic classifiers: three previously published and two newly proposed classifiers that were trained in a federated environment, and an additional “full” reference classifier that was trained with unconstrained data access.

Five of the classifiers are based on logistic regression, and the respective formulas are reported in Table 1. The remaining one is based on a federated implementation of the random forest method.

All these classifiers were obtained training different algorithms on a small subset of genes (*n* = 262) extracted from the full list (*n* = 13,041), using the volcano-plot-based feature selection function we implemented in VisualSHIELD (Supplementary Figure S3).

The “full” model is the classifier that one would expect to obtain after training a traditional logistic regression model on the small subset of genes. Since the federated GLM implementation available in DataSHIELD prevented us from training the model because of privacy constraints, we resorted to training it on a single machine with full access to a local copy of the data. The resulting “full” reference model was used for comparison purposes and ended up including 17 genes and 8 gene–gene interaction terms as predictors.

We then progressively restricted the “full” model by removing less relevant terms, namely, the terms with lower beta values or higher p-values, until the new model was deemed safe and acceptable for training by DataSHIELD. The formula for the restricted model can be found in Table 1 and ended up containing 10 genes and 7 gene–gene interaction terms as predictors.

This incident shows that in order to preserve privacy, federated alternatives of canonical algorithms are not always able to reproduce the analyses that would be possible in an unrestricted environment. One might argue that a federated analysis carried out using tools such as DataSHIELD compensates for this penalty by enabling access to a potentially larger selection of datasets that would be possible without privacy guarantees.

**FIGURE 3:**
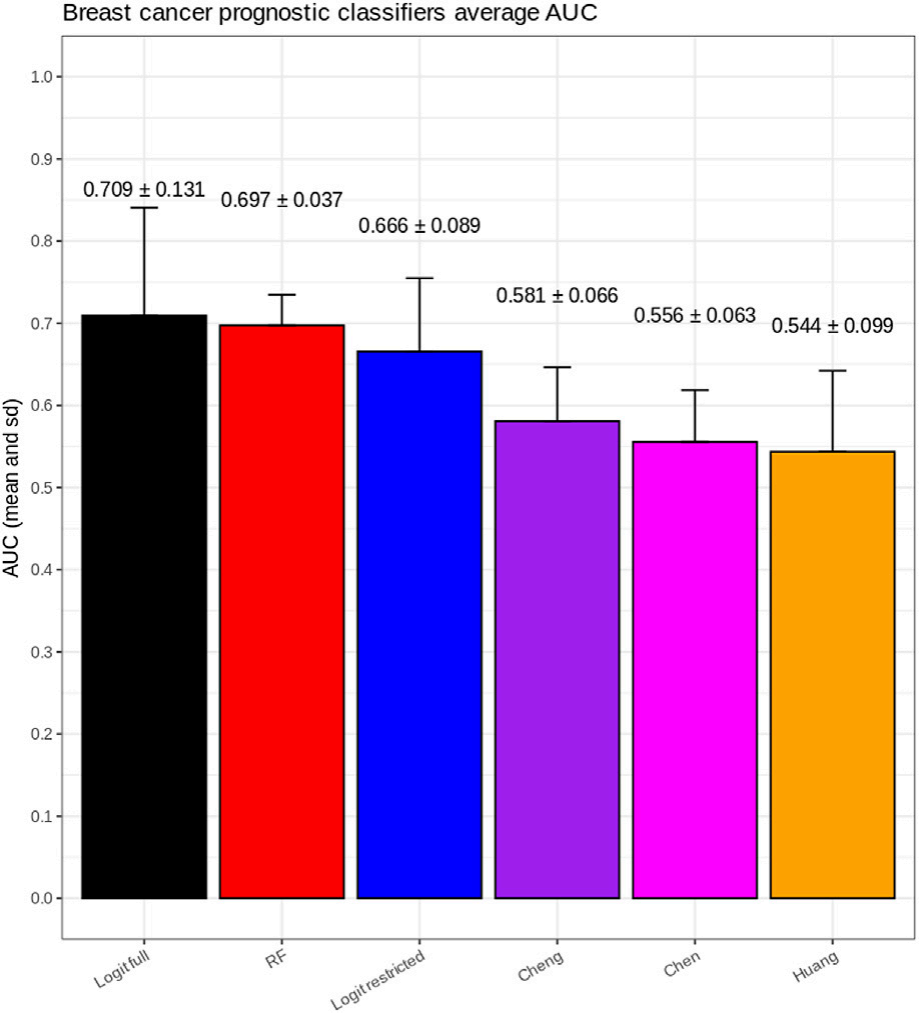
Average and standard deviations of the AUC for every model considered. Logit full was trained with full data access, while the others were trained in the privacy-preserving environment provided by VisualSHIELD. Although the full model has an average AUC greater than RF, the latter has a smaller standard deviation.

The ROC curves for each model in each of the five train/test rounds are shown in Figure 2, while the average and standard deviations of the AUC for every model considered are shown in Figure 3.

These results show a consistently superior performance of our newly proposed models, in particular RF, compared to the published breast cancer prognostic classifiers on unseen datasets.

Another observation is that, as expected, the unconstrained version of the logistic regression classifier (“full logit”) outperforms the federated version (“restricted logit”) but only by a modest 4% on average.

Finally, we sought to characterize the impact of distributed implementation of the linear model fitting algorithm. To compare the performance of the federated *versus* the canonical GLM algorithm, we trained the restricted model with full data access.

After performing the same cross-validation scheme and averaging the AUCs obtained, we found that the restricted model built with full data access shows the same performance as the restricted model trained in the federated environment (data access average AUC: 0.667, with standard deviation 0.091; federated average AUC: 0.666, with standard deviation 0.091). This result supports the conclusion that when not limited by disclosure risks, statistical models trained in a federated environment have the same predictive power of equivalent models trained with full data access, a testament to the quality of the implementation of the federated GLM in DataSHIELD.

## Discussion

We systematically evaluated three previously published and three newly proposed breast cancer relapse or cancer-free survival classifiers and found that our models outperform previous state-of-the-art classifiers.

Even if our models show superior performance, it is not clear whether they reach the level required for clinical use. To make things worse, our experiments show that the published results can be hard to replicate. To address both issues, it has been shown that a substantially larger sample size is required (Ein-Dor et al., 2006; Forouzandeh et al., 2022). An important contribution of our work is to make available a tool that facilitates experiments needing access to large collections of data.

Our analysis shows a limit of statistical modeling in a federated environment: some models cannot be trained because they are disclosive of individual-level data in some datasets. Training such a model in a canonical full-data access setting with the standard *glm* command led to a marginally higher average AUC and thus better average prediction performances.

On the other hand, training the same privacy-preserving model both in a federated and non-federated setting showed comparable performances, supporting the notion that when not limited by disclosure risks, statistical models trained in a federated environment have the same predictive power of equivalent models trained with full-data access.

From a biological point of view, limiting the number of independent variables in the statistical model is not necessarily an unsurmountable issue since a model with many variables is often of little practical applicability in a clinical setting.

From an analytical point of view, this may be viewed as an issue since it is limiting the choice of models that can be trained. We argue that this is an unavoidable trade-off of the DataSHIELD framework, ideally compensated by the larger choice of the federated dataset it allows access to.

One interesting question to address is whether privacy is too restrictive for medical studies. Based on our results, we conclude that the smaller choice of the GLM trainable in a privacy-preserving environment is not necessarily damaging as we observe a relatively small performance penalty. Additionally, alternative approaches such as random forest can provide better overall results.

An open issue is the potential bias introduced by the reduction in the overall availability of data for research due to the privacy burden, which might disproportionally affect under-represented patient groups and/or diseases. We believe that the ongoing development of tools such as VisualSHIELD will ease such concerns by reducing the cost and complexity of compliance to privacy regulations.

DataSHIELD natively includes several federated algorithms, and other authors have proposed additional federated algorithms as independent R packages. Furthermore, canonical non-federated R packages can be used to analyze and plot federated results (Paluszynska et al., 2020). It should be noted that the DataSHIELD-federated analysis framework is by no means specific to gene expression data; it can be applied to virtually to any type of biomedical data that can be organized in a tabular format.

These tools collectively expand the possibility to derive insights from the federated analyses but require R proficiency to be set up and effectively used.

VisualSHIELD is an attempt to address these concerns with an open-source, customizable, and modular Shiny module. It integrates additional functionalities and a set of standardized, ready-to-use Docker containers. We believe this is another step toward reproducible federated data analysis, and to encourage this trend, we make VisualSHIELD available as an open-source tool.

## Data Availability

The datasets presented in this study can be found in online repositories. The names of the repository/repositories and accession number(s) can be found in the article/Supplementary Material.

## References

[B1] AffyMetrix Inc (2023). AffyMetrix datasheet. Santa Clara, CA: AffyMetrix Inc. Available at: https://assets.thermofisher.com/TFS-Assets/LSG/brochures/hgu133arrays_datasheet.pdf.

[B2] Allen D. M. (1974). "The relationship between variable selection and data agumentation and a method for prediction." 10.2307/1267500

[B3] Biondo S. Ramos E. Deiros M. Martí Ragué J. De Oca J. Moreno P. (2000). Prognostic factors for mortality in left colonic peritonitis: a new scoring system. J. Am. Coll. Surg. 191, 635–642. 10.1016/s1072-7515(00)00758-4 11129812

[B4] Bonnett L. J. Snell K. I. E. Collins G. S. Riley Richard D. (2019). Guide to presenting clinical prediction models for use in clinical settings. BMJ 365, l737. 10.1136/bmj.l737 30995987

[B5] Chen T.-H. Wei J.-Ru Lei J. Chiu J.-Y. Shih K.-H. (2021). A clinicogenetic prognostic classifier for prediction of recurrence and survival in asian breast cancer patients. Front. Oncol. 11, 645853. 10.3389/fonc.2021.645853 33816299 PMC8010242

[B7] Data sharing in the age of deep learning (2023). Data sharing in the age of deep learning. Nat. Biotechnol. 10.1038/s41587-023-01770-3 37020134

[B6] Doiron D. Marcon Y. Fortier I. Burton P. Ferretti V. (2017). Opal and Mica: open-source software solutions for epidemiological data management, harmonization and dissemination. Int. J. Epidemiol. 46 (5), 1372–1378. 10.1093/ije/dyx180 29025122 PMC5837212

[B8] Dragan I. Sparsø T. Kuznetsov D. Slieker R. Ibberson M. (2020). dsSwissKnife: an R package for federated data analysis. bioRxiv. 10.1101/2020.11.17.386813

[B9] Edgar R. Domrachev M. Lash A. E. (2002). Gene Expression Omnibus: NCBI gene expression and hybridization array data repository. Nucleic Acids Res. 30 (1), 207–210. 10.1093/nar/30.1.207 11752295 PMC99122

[B10] Ein-Dor L. Or Z. Domany E. (2006). Thousands of samples are needed to generate a robust gene list for predicting outcome in cancer. PNAS 103, 5923–5928. 10.1073/pnas.0601231103 16585533 PMC1458674

[B11] Forouzandeh A. Rutar A. Kalmady S. K. Russell G. (2022). Analyzing biomarker discovery: estimating the reproducibility of biomarker sets. PLOS ONE 17, e0252697. 10.1371/journal.pone.0252697 35901020 PMC9333302

[B12] Gaye A. Marcon Y. Isaeva J. LaFlamme P. Turner A. Jones E. M. (2014). DataSHIELD: taking the analysis to the data, not the data to the analysis. Int. J. Epidemiol. 43, 1929–1944. 10.1093/ije/dyu188 25261970 PMC4276062

[B13] Hastie T. Tibshirani R. Friedman J. (2017). The elements of statistical learning. Available at: https://hastie.su.domains/ElemStatLearn/.

[B14] Ho T. K. (1995). “Random decision forests,” in Proceedings of the 3rd International Conference on Document Analysis and Recognition, Montreal, Quebec, Canada, August 14 1995 to August 16 1995. 10.5555/844379.844681

[B15] Hocking R. R. (1976). A biometrics invited paper. The analysis and selection of variables in linear regression. Biometrics 32, 1. 10.2307/2529336

[B16] Hosmer D. W. Stanley L. Sturdivant R. X. (2013). Applied logistic regression. New Jersey, United States: Wiley. 10.1002/9781118548387

[B17] Huang C.-C. Tu S.-H. Lien H.-H. Jeng J.-Y. Huang C.-S. Huang C.-J. (2013). Concurrent gene signatures for han Chinese breast cancers. PLOS ONE 8, e76421. 10.1371/journal.pone.0076421 24098497 PMC3789693

[B18] Kologlu M. Elker D. Altun H. Sayek I. (2001). Validation of MPI and PIA II in two different groups of patients with secondary peritonitis. Hepatogastroenterology 48 (37), 147–151. 11268952

[B19] Laurynas K. Gupta S. Purvesh K. (2022). Increasing reproducibility, robustness, and generalizability of biomarker selection from meta-analysis using Bayesian methodology. PLOS Comput. Biol. 18, e1010260. 10.1371/journal.pcbi.1010260 35759523 PMC9269905

[B20] Marcon Y. Bishop T. Avraam D. Escriba-Montagut X. Ryser-Welch P. Wheater S. (2021). Orchestrating privacy-protected big data analyses of data from different resources with R and DataSHIELD. PLOS Comput. Biol. 17 (3), e1008880. 10.1371/journal.pcbi.1008880 33784300 PMC8034722

[B21] Merkel D. (2014). Docker: lightweight linux containers for consistent development and deployment. Linux J. 10.5555/2600239.2600241

[B22] Moons K. G. M. Altman D. G. Vergouwe Y. Royston P. (2009). Prognosis and prognostic research: application and impact of prognostic models in clinical practice. BMJ 338, b606. 10.1136/bmj.b606 19502216

[B23] Nelder J. Wedderburn R. (1972). Generalized linear models. J. R. Stat. Soc. 135, 370. 10.2307/2344614

[B24] Pagès H. Carlson M. Falcon S. Li N. (2023). AnnotationDbi: manipulation of SQLite-based annotations in bioconductor. Available at: https://bioconductor.org/packages/AnnotationDbi.

[B25] Paluszynska A. Biecek P. Jiang Y. (2020). randomForestExplainer: explaining and visualizing random forests in terms of variable importance. Available at: https://cran.r-project.org/package=randomForestExplainer.

[B26] Pinart M. Nimptsch K. Bouwman J. O Dragsted L. Yang C. De Cock N. (2018b). Joint data analysis in nutritional epidemiology: identification of observational studies and minimal requirements. J. Nutr. 148 (2), 285–297. 10.1093/jn/nxx037 29490094

[B27] Pinart M. Nimptsch K. Bouwman J. O Dragsted L. Yang C. De Cock N. (2018a). Joint data analysis in nutritional epidemiology: identification of observational studies and minimal requirements. J. Nutr. 148, 285–297. 10.1093/jn/nxx037 29490094

[B28] Powers D. M. W. (2008). Evaluation: from precision, recall and F-factor to ROC, informedness, markedness & correlation. Available at: https://www.researchgate.net/publication/228529307_Evaluation_From_Precision_Recall_and_F-Factor_to_ROC_Informedness_Markedness_Correlation.

[B29] Rstudio, Inc (2013). Easy web applications in R. Available at: http://www.rstudio.com/shiny/.

[B30] Skye Hung-Chun C. Tzu-Ting H. Yu-Hao C. Tee Benita K. T. Horng C.-F. Wang Y. A. (2017). Validation of the 18-gene classifier as a prognostic biomarker of distant metastasis in breast cancer. PLOS ONE 12, e0184372. 10.1371/journal.pone.0184372 PMC5590926 28886126

[B31] Stone M. (1974). Cross-validatory choice and assessment of statistical predictions. J. R. Stat. Soc. 36, 111–133. 10.1111/j.2517-6161.1974.tb00994.x

[B36] Tomasoni D. Lombardo R. (2024). cosbi-research/VisualSHIELD: First Public Release (1.0). Zenodo. 10.5281/zenodo.10523026

[B32] Vickers A. J. Elkin E. B. (2006). Decision curve analysis: a novel method for evaluating prediction models. Med. Decis. Mak. 26 (6), 565–574. Sage Journals - MDM. 10.1177/0272989X06295361 PMC2577036 17099194

[B33] Vitali F. Lombardo R. Rivero D. Mattivi F. Franceschi P. Bordoni A. (2018). ONS: an ontology for a standardized description of interventions and observational studies in nutrition. Genes & Nutr. 13, 12. 10.1186/s12263-018-0601-y PMC5928560 29736190

[B34] Wolfson M. Wallace S. E. Masca N. Rowe G. Sheehan N. A. Ferretti V. (2010). DataSHIELD: resolving a conflict in contemporary bioscience - performing a pooled analysis of individual-level data without sharing the data. Int. J. Epidemiol. 39, 1372–1382. 10.1093/ije/dyq111 20630989 PMC2972441

[B35] Xavier M. E. Yannick M. Demetris A. Banerjee S. Bishop T. R. P. Burton P. (2022). ShinyDataSHIELD—an R Shiny application to perform federated non-disclosive data analysis in multicohort studies. Int. J. Epidemiol. 10.1093/ije/dyac201

