# Supplementary figures and images for: Strengths and limitations of non-disclosive data analysis: a comparison of breast cancer survival classifiers using VisualSHIELD
Source: Front Genet. 2024 Jan 29;15:1270387. doi: 10.3389/fgene.2024.1270387 (PMC10859452; doi:10.3389/fgene.2024.1270387)

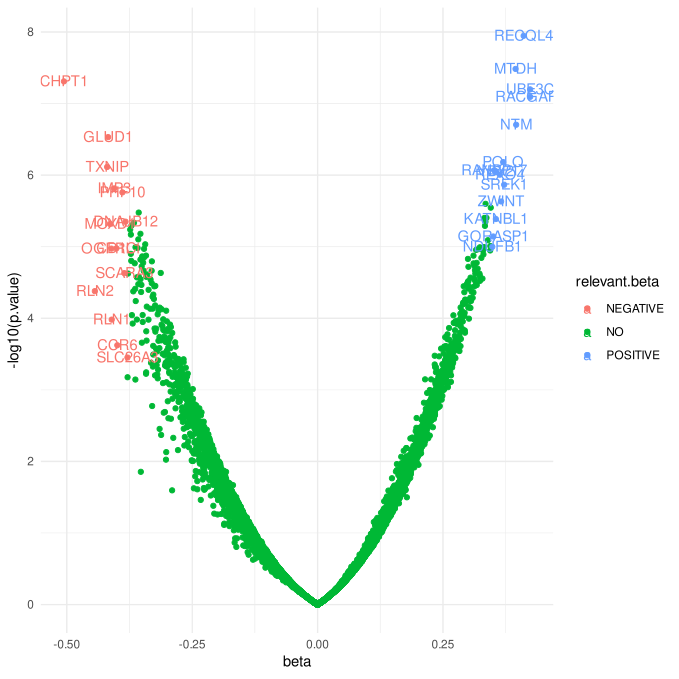

Supplement: Supplementary file 1 [file Image3.TIFF]

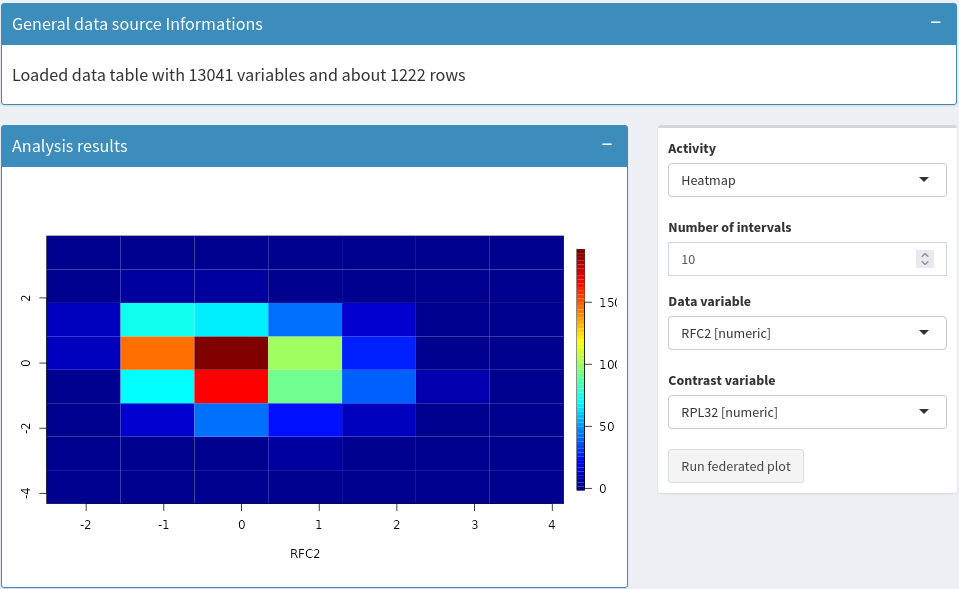

Supplement: Supplementary file 2 [file Image1.TIFF]

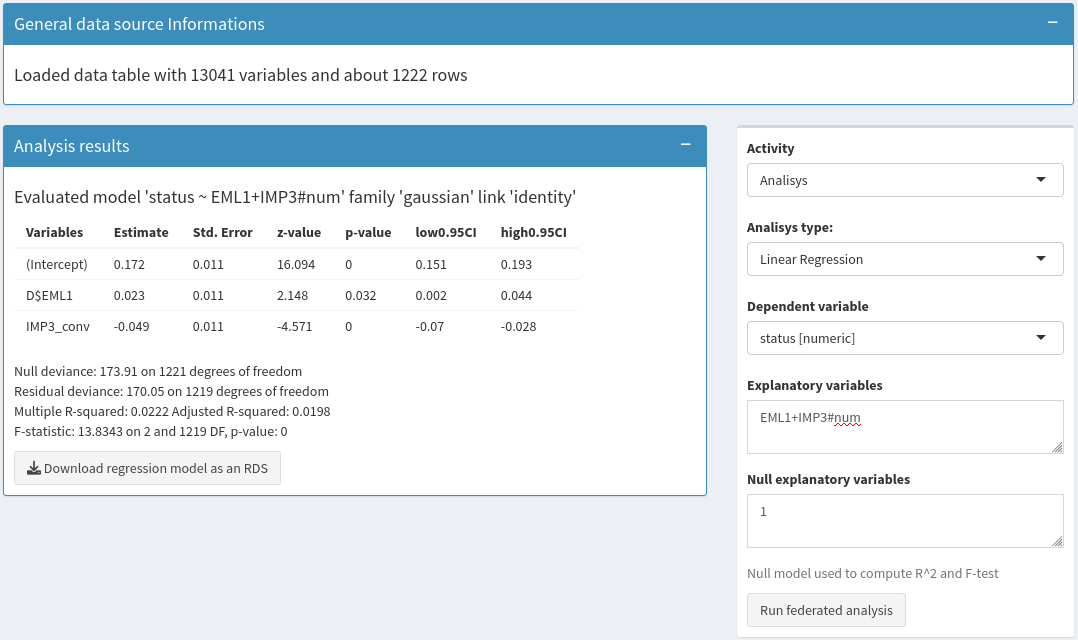

Supplement: Supplementary file 3 [file Image2.TIFF]
